# Enhancing Phenolic Compound Recovery from Grape Pomace Residue: Synergistic Approach of Ultrasound- and Enzyme-Assisted Extraction

**DOI:** 10.1021/acsomega.5c01321

**Published:** 2025-05-30

**Authors:** Natalia Stanek-Wandzel, Magdalena Zarębska, Tomasz Wasilewski, Zofia Hordyjewicz-Baran, Alicja Krzyszowska, Katarzyna Gębura, Magdalena Tomaka

**Affiliations:** † Institute of Heavy Organic Synthesis “Blachownia”, 556175 Łukasiewicz Research Network, Energetykow 9, 47-225 Kedzierzyn-Kozle, Poland; ‡ Faculty of Applied Chemistry, Casimir Pulaski Radom University, Chrobrego 27, 26-600 Radom, Poland

## Abstract

The objective of this study was to develop and optimize a novel ultrasound- and enzyme-assisted extraction (UEAE) method for isolating phenolic compounds, both free and cell-wall-bound, from red (RGP) and white (WGP) grape pomace. The research focused on evaluating the effects of key process parameters, including the composition and concentration of the enzyme mixture (pectinase, cellulase, and hemicellulase), pH, hydrolysis time, and sonication duration, on extraction efficiency and the chemical profile of the recovered phenolics. A specific aim was to compare the performance of the optimized UEAE method with solid–liquid extraction (SLE), ultrasound-assisted extraction (UAE), and enzyme-assisted extraction (EAE) in terms of both the quantity and diversity of phenolic compounds extracted. The results demonstrated that applying ultrasound after enzymatic hydrolysis significantly enhanced the release of the phenolic compounds. The UEAE method proved especially effective in extracting phenolic acids, such as gallic, caffeic, ferulic, and *p*-coumaric acids, with gallic acid content in red grape pomace reaching 431 mg/100 g and that in *p*-coumaric acid reaching 138 mg/100 g. Additionally, flavonoids, including quercetin, kaempferol, luteolin, vanillin, and *trans*-resveratrol as well as anthocyanins such as malvidin-3-glucoside and cyanidin chloride, were recovered in higher concentrations using the combined method, indicating its broad extraction capability. The highest total phenolic content (TPC) and antioxidant activity (measured via DPPH and ABTS assays) were observed when ultrasound treatment followed 60 min of enzymatic hydrolysis. This enhancement is attributed to the mechanical disruption of plant structures by ultrasound, which promotes the release of phenolics still bound within the cellular matrix. Overall, the optimized UEAE method proved to be an efficient and versatile approach for maximizing the recovery of bioactive compounds from grape pomace.

## Graphical abstract

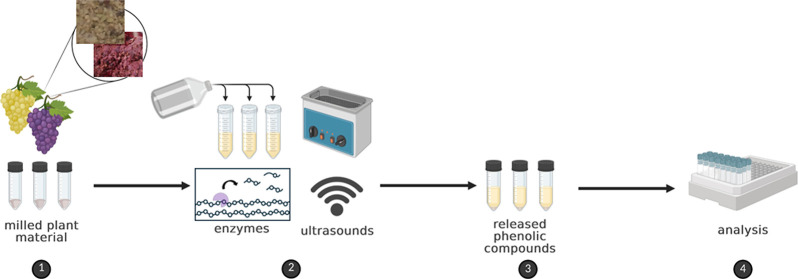

## 1 Introduction

Due to their health-promoting properties, plant extracts have been eagerly used by mankind for centuries in medicine, pharmacy, cosmetic, and food industries.^1^ Great importance is attached to the efficient extraction of active metabolites from plant material.^2,3^ However, some natural mechanical barriers impede the process, regardless of the extraction method. Many cell wall constituents, such as lignins, celluloses, pectins, or certain proteins, confer strength to the cells but also present obstacles during the extraction of bioactive components from the matrix. Lignins are hydrophobic and highly resistant to enzymatic and chemical degradation, cellulose forms crystalline microfibrils stabilized by strong hydrogen bonds, and pectins can create gel-like networks that entrap other molecules. In addition, some structural proteins can bind bioactive compounds, which limits the accessibility of these compounds to solvents and enzymes.^4^

An example of such bioactive components are phenolic compounds, found in plant material in various forms: as free (unbound) phenolics, as water-soluble glycosides, and as bound phenolic compounds, covalently linked to cell wall polymers (via ester or ether bonds), which makes them insoluble in water and inaccessible to conventional extraction methods.^5^ Bound phenols comprise an average of 24% of all of the phenolic compounds present in various plant matrices. This average, however, varies depending on the type of plant material and the specific tissue analyzed (e.g., peel, seed, leaf, or root).^5,6^ In fruit, insoluble, bound phenolic compounds account for between 6.5% and even 76%, as in the case of cranberry pomace.^5,7^ de Camargo et al. reported that bound phenolic compounds constitute the largest fractions in grape byproducts, being more effective as antioxidants than free phenolic fractions.^8^

Despite the growing interest in phenolic compounds, most extraction methods used in both industry and research continue to focus predominantly on the free fraction, overlooking the substantial proportion of phenolics in the bound form. This leads to under-utilization of the full antioxidant potential of plant materials. This creates a well-recognized research gap: the lack of efficient, scalable, and sustainable extraction strategies capable of releasing both free and bound phenolic fractions, especially from food industry byproducts such as grape pomace. Grape pomace is a particularly valuable source due to its high content of phenolic compounds,^9,10^ but current industrial processes do not adequately recover its full bioactive potential.

Extraction is a process that allows the separation of selected substances from a mixture by dissolving them in an appropriately chosen solvent and then separating them from the rest of the sample components. The effectiveness of extracting a compound from a plant material largely depends on the nature of the solvent used. When selecting a solvent for the extraction of phenolic compounds, its chemical properties such as polarity, acidity, and ability to form hydrogen bonds must be considered.^11^ Polar solvents such as methanol, ethanol, and their aqueous mixtures with acids such as hydrochloric, acetic, or formic acid are most commonly used for this purpose.

Thus, significant research effort has been devoted to developing extraction strategies capable of releasing both free and bound phenolics from plant tissues.^12–14^ However, they fail to break the covalent bonds between phenolics and cell wall components, leaving the bound fraction unrecovered. This is why traditional techniques result in incomplete phenolic recovery. These methods are mainly effective in extracting the free phenolic forms. However, releasing bound phenolics requires the application of additional physical or chemical factors.^15^

Classical techniques such as acidic or alkaline hydrolysis are increasingly being replaced by modern extraction methods. Acid hydrolysis involves treating plant extracts with inorganic acids (e.g., HCl or H_2_SO_4_), often under reflux and elevated temperatures.^16^ While it effectively breaks glycosidic bonds, it does not cleave ester bonds and may lead to degradation of thermolabile phenolics.^17^ Alkaline hydrolysis, using sodium hydroxide solutions (1–4 M), can release phenolic acids bound via ester linkages but may also result in degradation of some compounds such as hydroxycinnamic acid derivatives.^7^

To overcome these limitations, advanced methods such as enzyme-assisted extraction and ultrasound-assisted enzymatic extraction have been developed to target bound phenolics.^18,19^ Enzyme-assisted extraction involves the use of hydrolytic enzymes, such as cellulase, hemicellulase, pectinase, and β-glucosidase, which catalyze the breakdown of cell wall polysaccharides and glycosidic linkages. This disintegrates cellular structures and increases the permeability of bioactive components from the extracted raw material. Enzymatic reactions occur efficiently at relatively low temperatures and moderate pH in a relatively short time (up to a few hours) and do not require expensive equipment. Moreover, mild operating conditions help to preserve the structural integrity of phenolic compounds, minimizing degradation or isomerization.^20,21^

Researchers are increasingly modifying enzymatic extraction by combining it with, among others, ultrasound-assisted extraction to efficiently extract target compounds from the matrix.^22^ In enzymatic extraction, enzymes are often unable to completely hydrolyze the plant cell wall. Furthermore, the release of high-molecular-weight polyphenolic compounds (e.g., condensed tannins and proanthocyanidins) may be limited due to their low solubility and strong interactions with cell wall polymers.^23^ The cavitation process induced by ultrasound, which physically breaks down the plant matrix, can facilitate the enzymatic reaction and release of bound forms of phenolic compounds. In addition, the use of ultrasounds increases the contact surface area between the solid and liquid phases, which translates into faster contact between enzyme and substrate and faster enzymatic reaction.^15^

Alternative innovative extraction techniques include microwave-assisted extraction (MAE), pulsed electric field (PEF) extraction, and ultrasound-assisted extraction. These modern methods accelerate extraction kinetics and improve the release of bound phenolics from the plant matrix. MAE is particularly effective for short-chain phenolics but not for polymeric ones such as tannins or anthocyanins due to thermal degradation.^24^ UAE, which utilizes acoustic cavitation, shortens the extraction time and increases the penetration of the solvent into the matrix, although free radicals generated during cavitation may affect sensitive compounds.^25^ PEF, in turn, applies short high-voltage pulses that disrupt cell membranes via electroporation, facilitating phenolic release without thermal damage or chemical contamination.^26^

Tchabo et al. studied the effect of combining these extraction methods on the extraction of phenolic compounds from mulberry must.^27^ They observed a higher extraction efficiency of UAEE, compared with ultrasonic-assisted extraction or EAE alone. Wu et al. investigated the enzymatic extraction of polysaccharides from pumpkins, both with and without the use of ultrasound. The combination of ultrasonic treatment and enzymatic action resulted in higher extraction efficiency compared to using enzymatic extraction or ultrasonic-assisted extraction alone.^28^ Similarly, recent research on Trapa quadrispinosa stems demonstrated that ultrasonic-assisted enzymatic extraction not only maximized phenolic yield but also enhanced antioxidant and antitumor activities, confirming the effectiveness of this hybrid technique in improving both extraction efficiency and bioactivity of plant-derived compounds.^29^ These findings support the idea that hybrid extraction techniques can significantly improve the recovery of phenolics from plant materials. Results from the extraction of bioactive compounds from plant materials show that the complementary approach offers more benefits than the use of a particular type of extraction independently. However, these unique extraction procedures have yet to be properly developed to achieve optimal conditions for their use and to produce the expected results. Among the most critical parameters requiring optimization are ultrasound duration and intensity, enzyme type and concentration, extraction temperature, pH, and solid-to-liquid ratio. Each of these factors can significantly influence the release and stability of phenolic compounds, and their synergistic effects must be carefully evaluated to maximize extraction efficiency.^22^

Therefore, the aim of this study was to develop and optimize a method combining enzyme-assisted extraction (using a mixture of cellulase, hemicellulase, and pectinase) and ultrasound-assisted extraction to efficiently release both free and bound phenolic compounds from red and white grape pomace. This research focuses specifically on phenolic compounds due to their high biological value and antioxidant potential. The combined extraction technique is expected to improve yield, reduce degradation, and support the sustainable valorization of grape industry byproducts.

## 2 Material and Methods

### 2.1 Chemicals and Reagents

LC–MS-grade methanol was obtained from J.T. Baker (Phillipsburg, NJ, USA). All standards used in the study were of analytical grade with a minimum purity of 99%. Sodium citrate and citric acid were supplied by Chempur (Piekary Śląskie, Poland). Ultrapure water, with a resistance of less than 18 MΩ cm, was generated using a Direct-Q water purification system.

The identification and quantification standards included syringic acid, vanillic acid, rutin, *trans*-ferulic acid, *p*-coumaric acid, kaempferol, caffeic acid, 3,4-dihydroxybenzoic acid, vanillin, luteolin, and kuromanin chloride, all sourced from Merck (Darmstadt, Germany); *trans*-resveratrol from LGC (Teddington, Middlesex, UK); quercetin, gallic acid, ABTS (2,20-azino-bis­(3-ethylbenzothiazoline-6-sulfonic acid) diammonium), and Trolox (6-hydroxy-2,5,7,8-tetramethylchromane-2-carboxylic acid) from POL-AURA (Zabrze, Poland); DPPH (2,2-diphenyl-1-picrylhydrazyl) from Sigma-Aldrich (Saint Louis, MO, USA); and malvidin-3-glucoside chloride from PHYTO LAB (Vestenbergsgreuth, Germany).

Additionally, enzymes used in the study—cellulase from Aspergillus niger (0.9 U/mg), hemicellulase from A. niger (1.5 U/mg), and pectinase from Aspergillus (0.5 U/mg)—were procured from Sigma-Aldrich (Saint Louis, MO, USA). The enzymatic activity was defined according to the manufacturer’s specifications, where 1 unit (U) corresponds to the release of 1 μmol of reaction product per hour under standard assay conditions.

### 2.2 Plant Material

The grape pomace samples were obtained from the Estro Vineyard in Ujazd, Poland. The red grape pomace (RGP) was derived from Léon Millot hybrid varieties (Millardet et Grasser 101 O.P. × Goldriesling × Vitis rupestris × Vitis riparia), while the white grape pomace (WGP) was obtained from Solaris hybrid varieties (Merzling × Geisenheim 6493). The grape pomace samples were mixed with dry ice in a laboratory knife mill (Cutter Mixer R5 Plus, Robot Coupe, Palinges, France), packed in polyethylene plastic bags, frozen, and stored at −20 °C for further analysis. The dry matter content of the grape pomace samples was determined using the thermogravimetric method (Mettler Toledo TGA2 Thermogravimetric Analyzer).

To prepare the sample, a 10 kg batch of grape pomace was thoroughly mixed to achieve consistency. From this mass, 10 portions of 50 g were taken from different areas within the pomace mass and then crushed and homogenized to form a representative sample of 500 g. A 0.4 g portion of the ground grape pomace was weighed and mixed with 20 mL of eluent.

### 2.3 Extraction Procedure

#### 2.3.1 Solid–Liquid Extraction

Solid–liquid extraction is a widely used method for the recovery of unbound phenolic compounds from grape pomace. In this process, 0.4 g of ground grape pomace powder was extracted with 20 mL of citrate buffer (pH 4.0 ± 0.1) at 30 °C for 1.5 h with shaking at 150 rpm (BioSan Environmental Shaker-Incubator ES-20/60). After extraction, the mixture was filtered through a 0.45 μm nylon syringe filter to remove the solids prior to analysis.

#### 2.3.2 Ultrasonic-Assisted Extraction

Ultrasound-assisted extraction was also investigated as a means to enhance the extraction of phenolic compounds from grape pomace. A similar procedure was followed, where 0.4 g of ground grape pomace powder was mixed with 20 mL of citrate buffer and extracted in an ultrasonic cleaner (Pulsonic Sonic-6D) at 30 °C for 1.5 h at 2 × 320 W. The ultrasonic frequency was set at 40 kHz. The extract was filtered (0.45 μm nylon syringe filter) and analyzed.

#### 2.3.3 Enzyme-Assisted Extraction

To improve the extraction efficiency, enzyme-assisted extraction was employed. The procedure was carried out using the optimum extraction conditions established after using the design of experiments (DoE), i.e., the appropriate concentration of each enzyme, pH of the buffer, and incubation temperature to obtain the highest concentration of total phenolic compounds and antioxidant activity (see Section 2.4). In this method, 0.4 g of ground grape pomace powder was mixed with an enzymatic solution containing cellulase (5 U/mL), hemicellulase (12.5 U/mL), and pectinase (8 U/mL) in 20 mL of citrate buffer (pH 4.0 ± 0.1). The mixture was incubated at 30 °C for 1.5 h with shaking (BioSan Environmental Shaker-Incubator ES-20/60). The extract was filtered (0.45 μm nylon syringe filter) and analyzed.

#### 2.3.4 Ultrasonic-Assisted Enzymatic Extraction

To further enhance the release of phenolic compounds, a combination extraction technique integrating ultrasound-assisted and enzyme-assisted extractions was applied. This approach leveraged the mechanical effects of ultrasound along with the targeted degradation of cell wall components by enzymes. The enzymatic step was conducted under the same optimized conditions as described above for EAE. Ultrasound treatment was applied in two stages: before and after enzyme incubation to assess the effect of ultrasound timing on extraction efficiency. Ultrasound-assisted extraction was performed at a frequency of 40 kHz and a total power of 2 × 320 W. Three different ultrasound exposure times—10, 30, and 60 min—were tested in preliminary trials to identify the most effective duration. Following ultrasound and enzymatic treatments, the extracts were filtered by using a 0.45 μm nylon syringe filter and analyzed.

### 2.4 Experimental Design

In this study, the effect of some variables on the enzyme-assisted extraction of phenolic compounds and antioxidant activity from red grape pomace was investigated using a statistical design of experiments approach (StatSoft 13.3 software). The extraction conditions were investigated for each enzyme separately, and the ratio of mass-to-eluent (0.4 g/20 mL) and extraction time (1.5 h) were determined.^30^ After the individual optimization, enzymatic extraction was performed using combinations of the enzymes: cellulase, hemicellulase, and pectinase (CHP). The variables tested were pH (4.0, 4.5, 5.0), temperature (30, 40, 50 °C), and mix of concentrations of cellulase (C) (5, 12.5, 20 U/mL), hemicellulase (H) (5, 12.5, 20 U/mL), and pectinase (P) (5, 12.5, and 20 U/mL). The design of experiments employed in this study involved 31 experiments at the center points, with the variables outlined in Table 1 (see Section 3.1). The experimental data were subjected to regression analysis and modeled by using a second-order polynomial equation. The empirical model is expressed as

1 $$y_{k}=\beta_{0}+\beta_{1}x_{i1}+\beta_{2}x_{i2}+...+\beta_{k}x_{ik}+\varepsilon_{i}$$

where *y*_*k*_ = response variable, *y*_1_ = TPC (mg GAE/100 g), *y*_2_ = DPPH (mg TE/100 g), and *x*_1_, *x*_2_, *x*_3_, *x*_4_, and *x*_5_ represent the coded independent variables for the concentration of each enzyme, pH, and extraction temperature. β_0_ is the model constant.

**Table 1:** Experimental Conditions Used in the Design of Experiments to Evaluate the Effect of Enzyme Concentrations, pH, and Temperature on the Extraction of Phenolic Compounds from Red Grape Pomace^a^

run order	cellulase (U/mL of RGP)	hemicellulase (U/mL of RGP)	pectinase (U/mL of RGP)	pH	*T* (°C)	TPC (mg GAE/100 g of RGP)	DPPH (mg TE/100 g of RGP)
1	5	5	5	5	30	1378	5138
2	5	5	5	4	50	1244	4311
3	5	5	20	4	30	1816	4676
4	5	5	20	5	50	1313	3751
5	5	20	5	4	30	1951	5182
6	5	20	5	5	50	1311	4387
7	5	20	20	5	30	1760	4704
8	5	20	20	4	50	1085	3596
9	20	5	5	4	30	1820	5164
10	20	5	5	5	50	1480	4152
11	20	5	20	5	30	1472	4374
12	20	5	20	4	50	1127	3641
13	20	20	5	5	30	1495	5104
14	20	20	5	4	50	1039	4030
15	20	20	20	4	30	1683	4492
16	20	20	20	5	50	1243	3699
17	5	12.5	12.5	4.5	40	1470	4510
18	12.5	12.5	12.5	4.5	40	1440	4574
19	12.5	5	12.5	4.5	40	1428	4607
20	12.5	20	12.5	4.5	40	1129	4112
21	12.5	12.5	5	4.5	40	1557	5309
22	12.5	12.5	20	4.5	40	1342	3986
23	12.5	12.5	12.5	4.5	30	1732	5163
24	12.5	12.5	12.5	4.5	50	1174	3925
25	12.5	12.5	12.5	4	40	1539	4623
26	12.5	12.5	12.5	5	40	1462	4895
27	12.5	12.5	12.5	4.5	40	1821	4869
28	12.5	12.5	12.5	4.5	40	1735	4747
29	12.5	12.5	12.5	4.5	40	1742	4951
30	12.5	12.5	12.5	4.5	40	1770	5028
31	12.5	12.5	12.5	4.5	40	1736	4696

a Abbreviations: RGP—red grape pomace; TPC—total phenolic content; DPPH—antioxidant activity; GAE—gallic acid equivalents; TE—Trolox equivalents.

### 2.5 Analysis of Grape Pomaces Extracts

#### 2.5.1 Determination of Phenolic/Bioactive Compounds by UHPLC–MS/MS

The filtered extract solutions were analyzed by using a high-performance liquid chromatograph (Sciex ExionLC AD, AB Sciex, Concord, ON, Canada). Chromatographic separation was performed using a Kinetex XB-C18 column (3.5 μm, 100 Å, 100 × 4.6 mm; Phenomenex) maintained at 30 °C. The mobile phase consisted of 0.5% (v/v) aqueous formic acid (solvent A) and methanol (solvent B). The gradient elution program was as follows: 15–50% B from 0.0 to 20.0 min, maintained at 50% B from 20.0 to 25.0 min, decreased from 50% to 15% B between 25.0 and 25.1 min, and then held at 55% B from 25.1 to 30.0 min. The flow rate was set at 0.5 mL/min, with an injection volume of 10 μL. Detection utilized a quadrupole mass spectrometer (4500 QTRAP, AB Sciex, Concord, ON, Canada) with an electrospray ionization source capable of operating in both negative and positive ion modes. The quantification process employed the multiple reaction monitoring (MRM) scan mode. The MS/MS transitions, spectrometer parameters, and detailed protocol were documented in a prior study.^30^ Data analysis was carried out using Analyst software, version 1.7.2.

#### 2.5.2 Determination of Total Phenolic Contents

The total phenolic content (TPC) of red and white grape pomace extracts was determined using the Folin–Ciocalteu assay.^31^ For red grape pomace, 80 μL of the extract was mixed with 200 μL of the Folin–Ciocalteu reagent, followed by the addition of 600 μL of sodium carbonate solution (20%, w/v). For white grape pomace, 160 μL of the extract was mixed with 200 μL of Folin–Ciocalteu reagent and 600 μL of sodium carbonate solution (20%, w/v). The reaction mixtures were incubated in the dark at room temperature for 120 min, and the absorbance was measured at 765 nm using a spectrophotometer (Spectrophotometer HP—Hewlett-Packard, type: 8452A, Palo Alto, CA, USA). The total phenolic content was expressed in milligrams of gallic acid equivalent per 100 g of dry weight of grape pomace.

#### 2.5.3 Determination of Antioxidant Properties

The antioxidant properties of the grape pomace extracts were evaluated by using two spectrophotometric methods. The radical scavenging activity of DPPH was measured according to the modified method reported by ref 32, while the scavenging activity of the ABTS radical was assessed using the modified method described by^33^. For the DPPH assay, 3 mL of a 0.1 mM DPPH methanolic solution was mixed with 80 μL of extract from red grape pomace and 160 μL of extract from white grape pomace. The reaction mixtures were incubated in the dark at room temperature for 30 min, and the absorbance was measured at 517 nm. Results were expressed as milligrams of Trolox equivalents (mg TE) per 100 g of dry weight. For the ABTS assay, the ABTS radical cation (ABTS^•+^) was generated by mixing 14 mM ABTS solution with 5 mM potassium persulfate and allowing the mixture to react in the dark for 20 h. Before use, the solution was diluted with methanol to obtain an absorbance of 0.750 ± 0.05 at 734 nm. Then, 2 mL of diluted ABTS^•+^ solution was mixed with 30 μL of extract from red grape pomace and 40 μL of extract from white grape pomace, incubated for 6 min at room temperature, and the absorbance was measured at 734 nm. Results were expressed as mg TE per 100 g of dry weight.

## 3 Results and Discussion

### 3.1 Optimization of Enzyme-Assisted Extraction Conditions

The goal of this study was to determine the best conditions for efficiently extracting bioactive compounds using enzyme-assisted extraction. For this purpose, the optimization of enzymatic extraction of phenolic compounds and antioxidant activity from grape pomace was conducted using a statistical design of experiments approach. Once the optimum conditions for each enzyme were determined,^30^ the three enzymes—cellulase, hemicellulase, and pectinase—were combined to maximize extraction efficiency. Key variables tested in the study were as follows: pH, temperature, and enzyme concentrations. A series of experiments was conducted to identify the optimal values for these parameters to maximize the extraction yield. The detailed setup of the experiments and the variables tested are summarized in Table 1, providing a comprehensive overview of the parameters explored for enzymatic optimization. Additionally, Figure 1 presents the data graphically by using response surface plots. The experimental results were analyzed to determine the optimal conditions for the enzyme mixture (cellulase: 5 U/mL, hemicellulase: 12.5 U/mL, pectinase: 8 U/mL; temperature = 30 °C; pH = 4), which were then applied to produce the corresponding grape pomace extracts.

**Figure 1:**
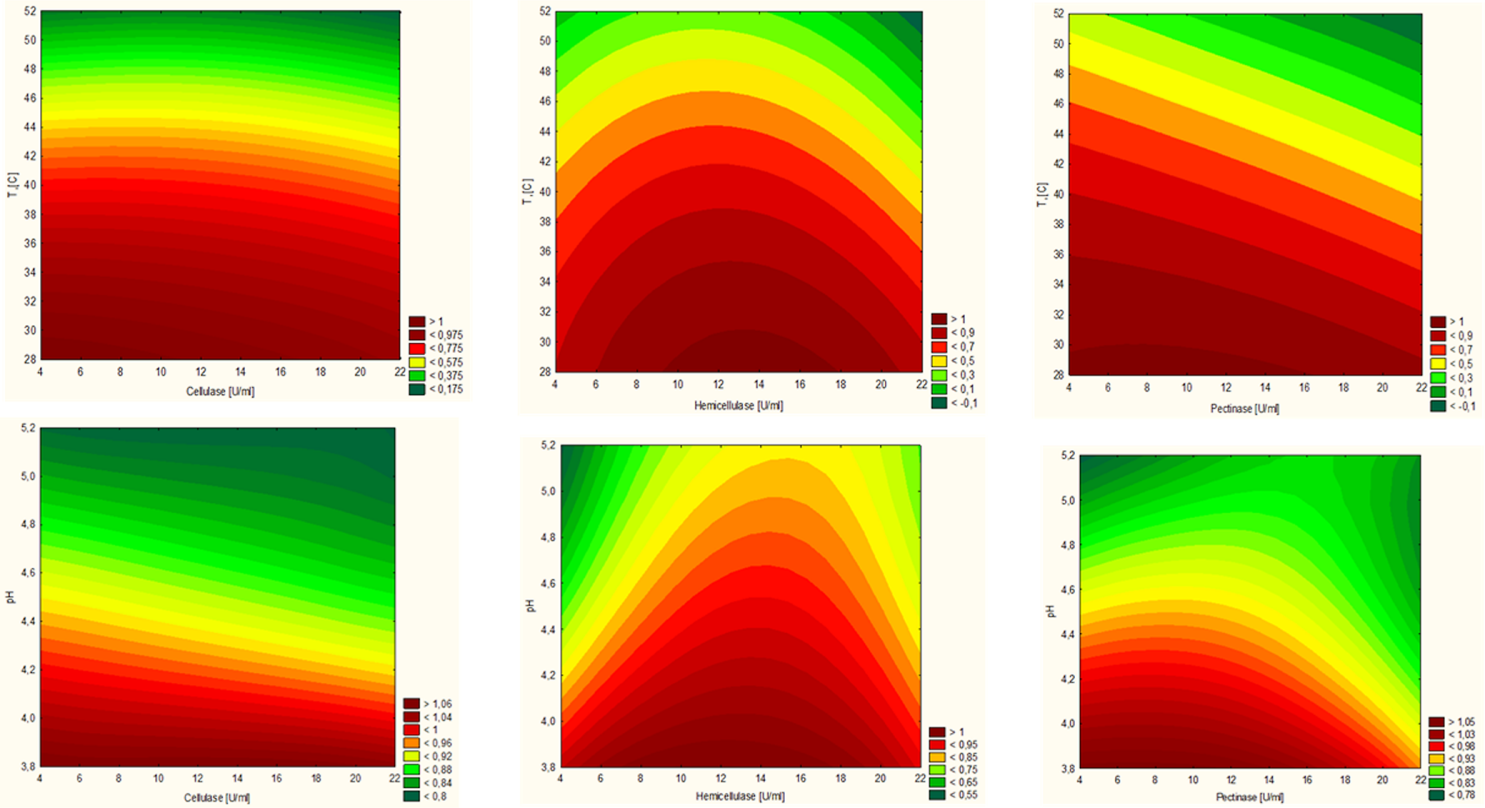
Response surface plots show the relationships between total phenolic content, antioxidant activity extracts, and reaction parameters: pH, temperature, and enzyme concentrations. Warmer colors (red/orange) indicate higher levels of extracted phenolics or antioxidant activity, whereas cooler colors (green) represent lower values. These visual cues help identify optimal extraction conditions.

The final predictive equation was determined based on the regression coefficients and variable values, including the cellulase concentration, hemicellulase concentration, pectinase concentration, pH, and temperature. Using this equation, the predicted total phenolic content was 2114 mg of GAE/100 g and the DPPH radical scavenging activity was 5316 mg of TE/100 g. These predicted values closely aligned with the observed values of 2095 mg of GAE/100 g (RSD = 0.6) and 5375 mg of TE/100 g (RSD = 0.8), respectively. This strong agreement between the predicted and experimental results confirms the reliability of the model for the optimization.

### 3.2 Phenolic Profile of Extracts

The aim of this study was to evaluate the efficiency of two extraction methods: enzyme-assisted extraction and ultrasound-assisted extraction, both individually and in combination, in extracting phenolic compounds from red and white grape pomace. As a control for enzyme-assisted extraction, solid–liquid extraction under the same conditions but without enzyme addition was conducted (control). The results presented in this study (Figures 2 and 3) indicated that enzyme-assisted extraction was a more efficient method for extracting phenolic acids, particularly gallic, *p*-coumaric, and caffeic acids, compared with ultrasound-assisted extraction. Interestingly, caffeic acid was extracted exclusively with enzyme-assisted extraction, suggesting a higher selectivity of this method for this compound. This observation may be attributed to the specific hydrolytic activity of enzymes (such as cellulases, pectinases, and hemicellulases), which facilitate the breakdown of complex cell wall polysaccharides and can enhance the release of phenolic acids embedded in the plant matrix.^34^ Caffeic acid rarely occurs in its free form in fruits, more often occurring as an ester with other phenols, such as chlorogenic acid (an ester of caffeic and quinic acids), or bound to the cell wall.^35^ Therefore, its release is more effectively achieved through enzymatic treatments that break down the cell wall structure and can liberate phenolic acids from conjugated or matrix-associated forms. In contrast, ultrasound lacks the biochemical specificity to cleave such bonds, potentially explaining the absence of caffeic acid in the UAE extracts. On the other hand, ultrasound-assisted extraction proved to be more effective for certain flavonoids, especially anthocyanins. It emerged as a gentler method compared to enzyme-assisted extraction, which, although effective in breaking down cell wall components, may expose anthocyanins to conditions that promote their degradation. In this study, both EAE and UAE were conducted in a citrate buffer at pH 4.0 ± 0.1, a temperature of 30 °C, and for 1.5 h. The EAE protocol additionally involved a combination of cellulase, hemicellulase, and pectinase. While these enzymes were selected to efficiently disrupt plant cell walls and the extraction environment, their activity may have led to the release of secondary metabolites or altered local pH or redox conditions, which could contribute to the degradation of anthocyanins. Anthocyanins are chemically unstable under mildly acidic conditions. At pH 4.0, anthocyanins exist near the equilibrium between the flavylium cation (colored) and other less stable or colorless forms, such as chalcones and pseudobases. The enzymatic environment in EAE may have favored shifts in this equilibrium, compromising the pigment’s integrity and color.^36^ In contrast, UAE employs physical disruption mechanisms, which avoid enzymatic side effects and help maintain the structural integrity and color stability of the anthocyanins. The improved extraction of delphinidin chloride under UAE conditions is likely a result of these nonenzymatic, purely mechanical processes, which are more compatible with the sensitive nature of these compounds. Chemically, delphinidin chloride is a polar, water-soluble anthocyanin with multiple hydroxyl groups, making it highly reactive and relatively unstable.^37^ As such, it is especially susceptible to degradation, reinforcing the importance of using nonenzymatic, gentle extraction techniques like UAE to preserve such sensitive compounds.

**Figure 2:**
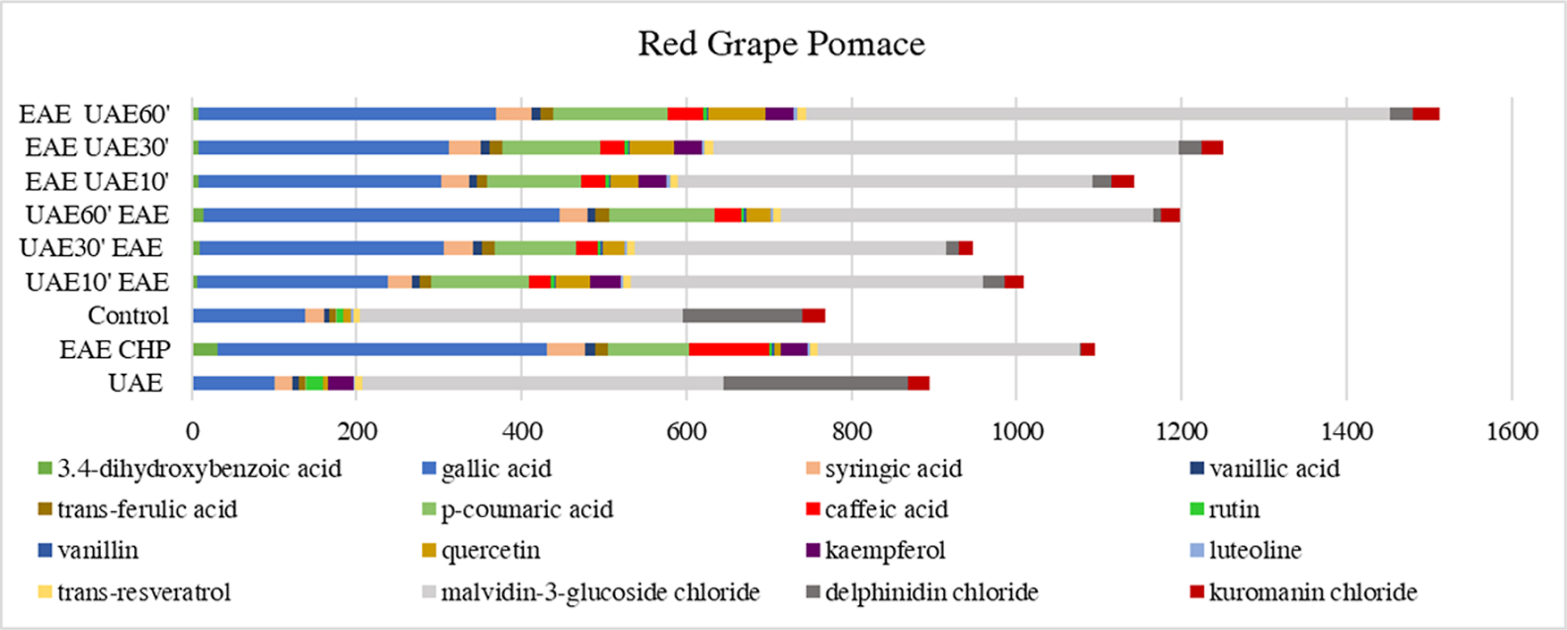
Proportion of phenolic compounds in red grape pomace extracts depending on the extraction method used. Abbreviations: UAE—ultrasound-assisted extraction; EAE CHP—enzymatic extraction using a mixture of cellulase, pectinase, and hemicellulase; control—solid–liquid extraction; UAE10′EAE—enzyme-assisted extraction preceded by 10 min of ultrasound-assisted extraction; UAE30′EAE—enzyme-assisted extraction preceded by 30 min of ultrasound-assisted extraction; UAE60′EAE—enzyme-assisted extraction preceded by 60 min of ultrasound-assisted extraction; EAE UAE10′—enzyme-assisted extraction followed by 10 min of ultrasound-assisted extraction; EAE UAE30′—enzyme-assisted extraction followed by 30 min of ultrasound-assisted extraction; EAE UAE60′—enzyme-assisted extraction followed by 60 min of ultrasound-assisted extraction.

**Figure 3:**
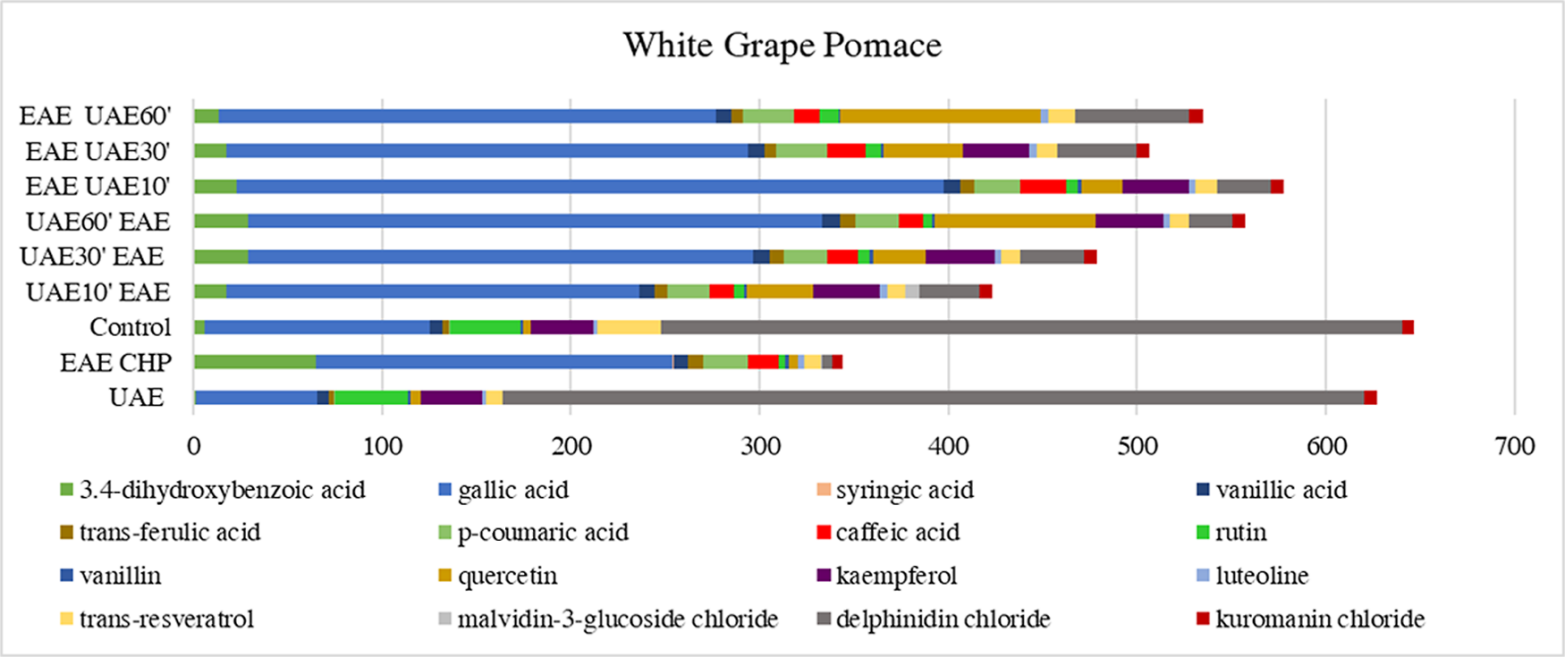
Proportion of phenolic compounds in white grape pomace extracts depending on the extraction method used. Abbreviations: UAE—ultrasound-assisted extraction; EAE CHP—enzymatic extraction using a mixture of cellulase, pectinase, and hemicellulase; control—solid–liquid extraction; UAE10′EAE—enzyme-assisted extraction preceded by 10 min of ultrasound-assisted extraction; UAE30′EAE—enzyme-assisted extraction preceded by 30 min of ultrasound-assisted extraction; UAE60′EAE—enzyme-assisted extraction preceded by 60 min of ultrasound-assisted extraction; EAE UAE10′—enzyme-assisted extraction followed by 10 min of ultrasound-assisted extraction; EAE UAE30′—enzyme-assisted extraction followed by 30 min of ultrasound-assisted extraction; EAE UAE60′—enzyme-assisted extraction followed by 60 min of ultrasound-assisted extraction.

These findings are consistent with reports in the literature highlighting the advantages of enzyme-assisted extraction in enhancing the recovery of bioactive compounds from various plant sources. For instance, polyphenol extraction from unripe apples using enzymes such as arabinose, cellulase, β-glucanase, hemicellulase, and xylanase increased *p*-coumaric, ferulic, and caffeic acid content by 8, 4, and 32 times, respectively.^38^ Durmus et al. noted that among the “non-extractable” phenolic fractions, enzyme-treated samples had the highest concentrations of all identified phenolics.^39^ Enzymatic treatment led to a two- to four-fold increase in phenolic content compared to heat or ultrasound treatment. Landbo and Meyer observed that enzymatic release of phenolics from blackcurrant pomace using protease and pectinase increased the overall polyphenol content, although anthocyanin extraction efficiency was lower.^40^ The authors attributed this reduced anthocyanin yield to the presence of β-glucosidase, β-galactosidase, or α-l-arabinofuranosidase in multienzyme preparations, which released anthocyanin sugars, forming unstable aglycones. Thus, it is evident that the extraction techniques employed significantly influence the recovery of specific phenolic classes, with ultrasound-assisted extraction being more effective for anthocyanins and enzyme-assisted extraction favoring the recovery of phenolic acids. This selectivity can be explained chemically by the different binding forms of phenolics in plant matrices; phenolic acids are often covalently or ester-linked to structural polymers like lignin or hemicellulose, requiring enzymatic breakdown,^41^ whereas anthocyanins are more likely to be present in vacuoles and released effectively via physical disruption.^42^

Moreover, combining enzyme and ultrasound extractions enhances phenolic compound recovery. For instance, in red pomace, gallic acid reached 431 mg/100 g after 60 min of ultrasound extraction prior to enzyme addition. For white pomace, the highest concentration of gallic acid was 374 mg/100 g. Compounds like *trans*-ferulic and *p*-coumaric acids also reached peak values with this combined method. Notably, *p*-coumaric acid concentration in red pomace reached 138 mg/100 g after 60 min of ultrasound followed by enzyme treatment. Flavonoids such as quercetin, vanillin, kaempferol, luteolin, and *trans*-resveratrol as well as anthocyanins like malvidin-3-glucoside chloride and cyanidin chloride also achieved maximum values with this method, suggesting that the combination of enzymes and ultrasound can effectively break down cell walls and release more bioactive compounds than individual methods. Interestingly, many of the phenolic compounds better extracted with combined UAE and EAE share common structural motifs such as hydroxyl groups on aromatic rings and glycosidic linkages (in the case of flavonoid glycosides). These features influence the solubility and bonding to cell wall components. Ultrasound breaks the physical barriers (cell walls and membranes), increasing accessibility, while enzymes then hydrolyze specific bonds (e.g., glycosidic or ester linkages), enabling more complete release. This explains the high yield of structurally diverse phenolics in the combined approach. Considering extraction time, the highest compound contents were achieved in extracts obtained during 1 h of ultrasound treatment. It was also observed that applying ultrasound after enzyme-assisted extraction was more effective in phenolic extraction than applying it before EAE. This can be explained by the fact that EAE initially loosens or hydrolyzes the complex matrix, releasing compounds that are still trapped within partially degraded cell wall fragments. Subsequent application of ultrasound further disrupts these fragments through cavitation and microstreaming, allowing a deeper penetration of the solvent and better mass transfer of released compounds. Conversely, applying UAE first may damage cells, but enzymes may become less effective afterward if their substrates are denatured or if cell fragments collapse and limit enzyme diffusion.

The synergistic effects of ultrasound and enzymes are attributed to the ability of ultrasound to disrupt plant cell walls, facilitating the penetration of enzymes and the release of bioactive compounds. This process is further enhanced by the violent agitation caused by ultrasound, which can positively impact enzyme activity. In addition to improved penetration, ultrasound may enhance enzyme–substrate interactions by increasing substrate accessibility and possibly slightly altering the enzyme conformation to increase catalytic activity. The physical and chemical synergy thus leads to more efficient degradation of plant matrix and higher yields of both free and bound phenolics.^43^

### 3.3 Total Phenolic Content and Antioxidant Activity of Extracts

The results of antioxidant capacity measurements (using the DPPH and ABTS methods) and the total phenolic content of extracts obtained from red and white grape pomace are presented in Table 2. Extracts from red grape pomace showed higher antioxidant activity after enzymatic extraction compared to that obtained with ultrasound-assisted extraction, while the lowest values were obtained for extracts produced using conventional solid–liquid extraction. Similar trends were observed in determining the total phenolic content. In the case of white grape pomace, the antioxidant activity of the extracts after ultrasound-assisted extraction was slightly higher than that after enzymatic extraction. Additionally, the highest values were obtained by using a combination of enzymatic and ultrasound-assisted extraction. It was observed that applying ultrasound both before and after enzymatic extraction contributed to an increase in the extraction efficiency. When considering the extraction time, extracts obtained through enzymatic extraction followed by 1 h of ultrasound treatment showed the highest antioxidant activity (red grape pomace: DPPH: 6512 mg of TE/100 g, ABTS: 10,433 mg of TE/100 g; white grape pomace: DPPH: 4264 mg TE/100 g, ABTS: 10,031 mg TE/100 g). Similar results were found when examining the total phenolic content, where the highest values were obtained for extracts prepared using a combination of enzymatic extraction and ultrasound, with the most effective method being ultrasound extraction applied after 60 min of enzymatic extraction (red grape pomace: 2794 mg GAE/100 g; white grape pomace: 1820 mg GAE/100 g).

**Table 2:** Total Phenolic Content and Antioxidant Activity of Extracts Based on the Extraction Method Used^a^

mg/100 g d.w.	UAE	EAE CHP	control	UAE10′ EAE	UAE30′ EAE	UAE60′ EAE	EAE UAE10′	EAE UAE30′	EAE UAE60′
Red Grape Pomace									
TPC	1781 ± 76	2095 ± 96	1714 ± 26	2361 ± 48	2291 ± 54	2568 ± 48	2558 ± 4	2676 ± 54	2794 ± 62
DPPH	4917 ± 13	5375 ± 17	4289 ± 52	5682 ± 82	5586 ± 47	6047 ± 27	6290 ± 63	6193 ± 3	6512 ± 58
ABTS	8509 ± 124	8467 ± 32	7658 ± 112	8757 ± 57	9019 ± 157	9688 ± 12	8853 ± 151	10,349 ± 160	10,433 ± 37
White Grape Pomace									
TPC	1288 ± 5	1399 ± 34	1070 ± 37	1497 ± 39	1632 ± 4	1669 ± 6	1726 ± 23	1724 ± 18	1820 ± 50
DPPH	3410 ± 1	3360 ± 29	2109 ± 56	3492 ± 40	3509 ± 5	3645 ± 6	3873 ± 14	4078 ± 42	4264 ± 13
ABTS	8153 ± 147	6762 ± 35	4901 ± 27	6820 ± 8	7482 ± 179	7502 ± 165	8875 ± 57	9031 ± 72	10,031 ± 83

a Abbreviations: UAE—ultrasound-assisted extraction; EAE CHP—enzymatic extraction using a mixture of cellulase, pectinase, and hemicellulase; control—solid–liquid extraction; UAE10′EAE—enzyme-assisted extraction preceded by 10 min of ultrasound-assisted extraction; UAE30′EAE—enzyme-assisted extraction preceded by 30 min of ultrasound-assisted extraction; UAE60′EAE—enzyme-assisted extraction preceded by 60 min of ultrasound-assisted extraction; EAE UAE10′—enzyme-assisted extraction followed by 10 min of ultrasound-assisted extraction; EAE UAE30′—enzyme-assisted extraction followed by 30 min of ultrasound-assisted extraction; EAE UAE60′—enzyme-assisted extraction followed by 60 min of ultrasound-assisted extraction.

## 4 Conclusions

The conducted study demonstrated that both enzyme-assisted extraction and ultrasound-assisted extraction significantly influence the phenolic profile and antioxidant activity of extracts obtained from red and white grape pomace. Enzymatic extraction proved particularly effective in releasing phenolic acids, such as gallic, *p*-coumaric, and caffeic acids. In contrast, ultrasound-assisted extraction better preserved sensitive compounds such as anthocyanins due to the mild physical conditions that limit their degradation. The highest total phenolic content and antioxidant activity were obtained by combining both methods, particularly when ultrasound extraction followed enzymatic treatment. This sequence allowed enzymes to initially loosen the plant matrix, followed by ultrasound, facilitating effective compound release. Extracts from red grape pomace exhibited a higher phenolic content and antioxidant activity compared to those from white grape pomace. However, in both cases, the highest values were recorded using enzymatic extraction supported by 60 min of ultrasound application. Specifically, the highest total phenolic content reached 2794 mg of GAE/100 g for red pomace and 1820 mg of GAE/100 g for white pomace. The corresponding antioxidant activities were as follows: DPPH: 6512 mg of TE/100 g (RGP), 4264 mg of TE/100 g (WGP); ABTS: 10,433 mg of TE/100 g (RGP), 10,031 mg of TE/100 g (WGP). In summary, the synergistic application of enzymatic and ultrasound-assisted extraction significantly enhances the recovery efficiency of bioactive compounds from plant materials, making this method particularly valuable in the context of valorizing byproducts from the wine industry.

## 5 Future Prospects

To further develop the potential of this extraction strategy, it would be worthwhile to expand the scope of research beyond grape pomace. A particularly interesting direction could involve applying this combined method to other byproducts of the wine industry, such as grape seeds, skins, or stems, which are also rich in phenolic compounds. Additionally, integrating enzyme-assisted extraction with other emerging green technologies, such as pulsed electric fields or microwave-assisted extraction, could lead to the development of more efficient and sustainable hybrid processes. These innovations may improve both the yield and selectivity of valuable bioactive compounds while minimizing environmental impact. Finally, considering the high antioxidant capacity of the obtained extracts, their use in the formulation of functional foods, dietary supplements, and natural cosmetic products appears highly promising. This aligns with current trends promoting the use of natural, health-enhancing, and ecofriendly ingredients in consumer products.

## Abbreviations

EAE: enzyme-assisted extraction
UAEE: ultrasound-assisted enzymatic extraction
GP: grape pomace
RGP: red grape pomace
WGP: white grape pomace
control: solid–liquid extraction
UAE: ultrasonic-assisted extraction
DoE: design of experiments
CHP: combinations of the enzymes: cellulase, hemicellulase, and pectinase
UHPLC–MS/MS: ultrahigh performance liquid chromatography with a tandem mass spectrometer
TPC: total phenolic contents
DPPH: 2,2-diphenyl-1-picrylhydrazyl
ABTS: 2,20-azino-bis­(3-ethylbenzothiazoline-6-sulfonic acid) diammonium
UAE10′EAE: enzyme-assisted extraction preceded by 10 min of ultrasound-assisted extraction
UAE30′EAE: enzyme-assisted extraction preceded by 30 min of ultrasound-assisted extraction
UAE60′EAE: enzyme-assisted extraction preceded by 60 min of ultrasound-assisted extraction
EAE UAE10′: enzyme-assisted extraction followed by 10 min of ultrasound-assisted extraction
EAE UAE30′: enzyme-assisted extraction followed by 30 min of ultrasound-assisted extraction
EAE UAE60′: enzyme-assisted extraction followed by 60 min of ultrasound-assisted extraction
GAE: gallic acid equivalents
TE: Trolox equivalents
